# The Effects of Hypertension on Cognitive Function in Children and Adolescents

**DOI:** 10.1155/2012/891094

**Published:** 2012-02-28

**Authors:** Stephen D. Cha, Hiren P. Patel, David S. Hains, John D. Mahan

**Affiliations:** ^1^Pediatric Nephrology, Nationwide Children's Hospital, Columbus, OH 43205, USA; ^2^The Ohio State University College of Medicine, Columbus, OH 43210, USA

## Abstract

Hypertension (HTN) is found in about 3-4% of the pediatric population with long-term risks of end organ damage if untreated or poorly controlled. Although children with HTN are being more frequently screened for end organ damage (i.e., LVH), the cognitive effects of HTN and methods to screen for cognitive dysfunction have not been extensively explored. In recent years, there have been a small number of studies that have provided important insights that can guide future research in this area. These studies show that HTN can be associated with headaches, restlessness, sleep disturbance, anxiety, depression, decreased attention, and also poor executive functioning. By increasing the utilization of cognitive tests in hypertensive children and adolescents, important cognitive defects secondary to HTN may be detected. More research is needed in the area, and the results of future studies could have far reaching implications for long-term outcomes in hypertensive children and adolescents.

## 1. Introduction

The prevalence of hypertension (HTN) is increasing in the pediatric population and is now up to 3-4% [1]. In adults, HTN is recognized to have multiple acute and chronic complications including negative effects on cognitive function. The long-term health risk for pediatric patients with HTN can have far reaching effects. Therefore, guidelines have been developed to assist in early and accurate diagnosis of HTN in order to minimize risk of end organ damage [2]. In pediatrics, hypertension is defined as a systolic and/or diastolic blood pressure (BP) ≥ 95th percentile for age, gender, and height on three or more readings [2]. It is becoming more common for practitioners to screen children with HTN for end organ damage, primarily left ventricular hypertrophy [3]. While there has been some focus on cognition in adult hypertensive patients, the effects of HTN on cognition in pediatric patients remain largely unexplored. More recently, a small number of studies on the cognitive effects of HTN in children have been published that provide important insights [3–5]. This paper highlights recently reported cognitive effects of pediatric HTN along with potential cognitive effects of long-term HTN.

## 2. Cognitive Development

Cognitive development is an active and ongoing process that is influenced by both internal and external stimuli from infancy to adolescence [6]. Developmental rates vary and are influenced by both innate capacity and external stimuli. With time and experience, the number and complexity of synaptic connections and neural plasticity promotes increasing cognitive sophistication. In infancy, the increase in neural connections results in rapid neurological growth and development. This is reflected in the achievement of developmental milestones in gross motor, fine motor, language, and social skills.

Developmental sequences are important in achieving full potential. Looking at one specific aspect of cognition, language, studies have shown that children with continued language disorders have more difficulties in adulthood compared to normal controls [7]. Children with language disorders often do not outgrow their impairments and if the language disorder is not corrected by school age, it can persist into early adulthood [7]. Language impaired adults have a higher rate of unemployment, lower socioeconomic status, and lower levels of education when compared to normal controls [7–9].

## 3. Pathophysiology of HTN on the Central Nervous System

### 3.1. Adults

**Figure 1:**
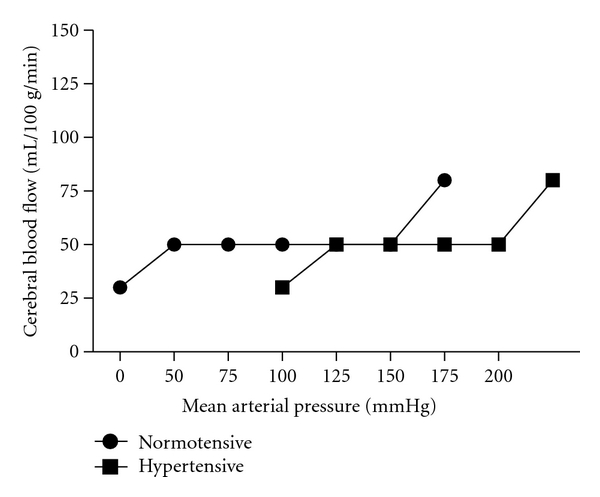
Connecting line graph of cerebral autoregulation in adult normotensive patients versus adult hypertensive patients.

In adults, the hypertensive effects on the brain are thought to be due to systolic blood pressure exceeding the autoregulatory mechanisms of the brain (Figure 1). This results in damage to small cerebral vessels that can lead to impaired autoregulation, lacunar infarcts, amyloid angiopathy, and even cerebral atrophy [10]. In adults, the amyloid angiopathy and cerebral atrophy can look similar to Alzheimer's disease [10]. These changes make it difficult to differentiate HTN that is associated with Alzheimer's disease from vascular dementia secondary to HTN.

Cognitive deficits in adults from HTN can be difficult to detect but may be divided into several domains including learning, memory, and attention [11]. The blood vessels in the prefrontal subcortical areas are often affected by severe HTN, which can affect the ability to make executive decisions (e.g., planning, attention, problem solving, verbal reasoning, etc.). Interestingly, Jennings et al. showed that adults with HTN had reduced cerebral blood flow to areas of the brain that are normally active during performance of cognitive tasks pertaining to memory [12, 13]. They also demonstrated that hypertensive individuals compensate for decreased cerebral blood flow by increasing blood flow to other areas of the brain. Over time, these cerebral changes can be manifested in cognitive or behavioral changes including sleep disturbances, concentration difficulties, and fatigue.

### 3.2. Pediatrics

**Figure 2:**
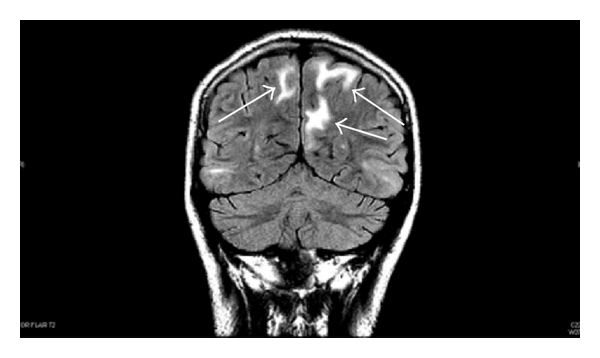
MRI T2 flair image demonstrating findings of posterior reversible encephalopathy syndrome involving bilateral parietal-occipital lobes, left greater than right (arrows).

Currently, most of the information on the pathophysiological effects of HTN on the pediatric central nervous system (CNS) relates to hypertensive encephalopathy [10]. When there is an acute severe elevation in blood pressure, children can present with seizures, ischemic, or hemorrhagic strokes, or even hypertensive encephalopathy [10]. This hypertensive emergency can be manifested as posterior reversible encephalopathy syndrome (PRES). Patients who develop PRES will commonly present with complaints of headache, visual changes, mental status changes, and can develop seizures. The diagnosis can be confirmed with the characteristic white matter changes seen on brain MRI in the posterior parietal and occipital regions (Figure 2). These changes can also be seen in the frontal lobes, basal ganglia, cerebellum, and brainstem. They are often seen symmetrically on imaging but asymmetrical lesions can be seen [14]. PRES is thought to result from a failure of the normal cerebral autoregulation in the vertebrobasilar vascular system leading to endothelial dysfunction, which ultimately results in localized areas of cerebral edema [14]. Once BP exceeds the upper limit of the autoregulatory system, the increased pressure is transmitted to the cerebral vasculature, especially the small vessels, leading to endothelial damage [10]. The disruption of the endothelium leads to increased vascular wall permeability, cell proliferation, and activation of the coagulation cascade, which ultimately leads to edema and tissue ischemia. It is possible that endothelial damage on a lesser scale than that seen in hypertensive encephalopathy affects cognitive function but this needs to be further examined. In addition, endothelial damage could be a forerunner for atherosclerosis in the future. In adults, carotid artery intima-media thickness (cIMT) is considered a surrogate marker for atherosclerosis [15]. cIMT has been shown to be increased in hypertensive pediatric patients suggesting early subclinical vascular injury and possible early atherosclerosis [16]. Thus, pediatric hypertension could be a risk factor for early atherosclerotic lesions. These lesions could potentially develop throughout the vasculature, but their effects on cerebral vasculature and its cognitive implications are unclear at this time.

The cognitive effects of HTN may also be in part related to inflammation. Lande et al. reported an association between elevated C-reactive protein (CRP) and elevated blood pressure in children and adolescents [17]. They showed that children and adolescents aged 8–17 years old participating in the Third National Health and Nutrition Examination Survey (NHANES III) with an elevated CRP level greater than 3 mg/L had higher systolic blood pressures when compared to those participants with a CRP less than or equal to 3 mg/L. In adults, an elevated CRP has been associated with cognitive impairments including in the area of visuospatial functioning [18]. Visuospatial skills are important for analyzing and understanding space which enables people to recreate the world through mental imagery.

## 4. Cognitive Impairments from HTN

### 4.1. Adults

Several behavioral and cognitive studies in adults have shown the negative effects of high blood pressure in the brain with resultant improvement after antihypertensive treatment. Shapiro et al. administered four behavioral tasks to 41 mildly hypertensive and 41 normotensive patients [19]. The hypertensive patients had significant behavioral impairment in three of the tasks: sensory-perceptual, cognitive, and psychomotor. They also showed that HTN seemed to preferentially affect behaviors that needed to be self-initiated. Miller et al. did a follow-up study where they tested the participants in the above study after 15 months of observation and treatment and compared them to normotensive individuals [20]. Those receiving antihypertensive therapy had a significant improvement of all behavioral-cognitive scores, which approached those of the normotensive control group.

Adult studies have demonstrated a variety of cognitive impairments associated with HTN. Essential hypertensive patients scored lower compared to normotensive patients on neuropsychological testing for speed, memory, spatial localization, simple motor skills, and learning [21]. The difference, however, was not apparent in everyday functioning capabilities.

Waldstein et al. showed that 20 untreated, mildly hypertensive adult males did poorer on learning and memory tests involving recall of visual stimuli compared to their normotensive controls [22]. A relationship between poor performance on cognitive tests and increased systolic blood pressure was found in 2727 men and women of ages 20 to 59 who participated in NHANES III [23]. Specifically, they found that patients with HTN did worse on the serial digit learning test, which is a measure of attention and concentration.

Elevated diastolic blood pressure in adults may be predictive of future cognitive function impairment [24]. A total of 999 men from the age of 50 through 70 were followed for up to 20 years. Cognitive function was assessed with the mini-mental state examination and trail-making test. The trail-making test is a neuropsychiatric test of visual attention and alternation of tasks. They found that low cognitive function was related to an elevated mean nocturnal diastolic blood pressure and nondipping pattern obtained from 24-hour ambulatory blood pressure monitoring. This relationship was greatest in those men who were not on antihypertensive treatment during the 20-year interim period.

It is known that adults with depression and anxiety have a higher prevalence of HTN, which may indicate that having HTN as a comorbidity may exacerbate psychiatric disorders [25]. The mechanism behind this association is still unclear. Of note, there was a study in adults with HTN who experienced improvement of their depression after administration of Captopril [26].

### 4.2. Pediatrics

In pediatrics, there have been several studies that demonstrated lower cognitive function in children and adolescents with hypertension. Lande et al. showed that participants aged 6–16 years old in the NHANES III who had a systolic blood pressure above the 90th percentile for height, age, and gender scored lower in short-term memory, attention, and concentration compared to their counterparts who had systolic blood pressure below the 90th percentile [3]. Those participants who were equal to or above the 95th percentile scored even lower when compared to the normotensive participants. The participants were asked to recite back random numbers in forward succession and then backwards. This study showed that, like in adults, psychological testing could be used to detect cognitive abnormalities in pediatric patients with HTN [21].

Lande et al. later reported the use of parental assessments to determine effects of HTN on behavior and executive functioning in children [27]. In this study, parents of untreated and newly diagnosed hypertensive patients aged 10–18 years old were given checklists to assess their children's behavior. Nonhypertensive children were used as a control group. The authors found that hypertensive children had more problems with behavior and mood disturbances such as anxiety, depression, and problems with attention compared to the control group. They also had poorer executive functioning when compared to the normotensive children. Executive functioning was measured by a behavior inventory, which was also reported by the parents. In their follow-up study, Lande et al. demonstrated improvement in executive functioning after 12 months of antihypertensive treatment. Executive functioning was defined with the same parental inventory method [28].

Adams et al. did a retrospective study of 201 patients age 10–18 years old who were referred to their institution for HTN evaluation [4]. One hundred patients had HTN and 101 were normotensive. Learning disabilities were more common in the patients who had a diagnosis of HTN. A learning disability was defined as having an individualized learning plan or section 504 plan in school. Whether the learning disabilities predated the diagnosis of HTN or were exacerbated by HTN was not evaluated by the design of this study.

An interesting study was reported by Krause et al. in which they surveyed parents of children who had been diagnosed with renovascular HTN at their institution [5]. Within the study period, 11 children had been diagnosed with renovascular HTN. Of those 11, 5 children had abnormal behavior prior to starting antihypertensive medications. The reported abnormal behaviors included restlessness, attention deficit disorder (ADHD), oppositional defiant disorder (ODD), temper tantrums, and sleep disturbances (Table 1). Of the 5 children with abnormal behavior, 3 had resolution of the behavior disturbances and the other 2 experienced great improvement after HTN treatment. This study supplements other clinical observations that young children may have behavioral disturbances secondary to HTN [27]. These behaviors may go unrecognized due to the difficulty of children to convey complaints in an effective manner. In a retrospective review, Croix and Feig reported that of those patients aged 7–18 years old who were referred to their institution for HTN and given that diagnosis, the hypertensive children reported more complaints of headache, daytime fatigue, difficulty with sleep initiation and chest, or abdominal pain compared to normotensive children[29]. The behavior changes may be attributed to nonorganic causes, and therefore HTN may go undiagnosed until the other nonorganic causes are excluded.

In children with mild-to-moderate chronic kidney disease (CKD), elevated blood pressure was associated with decreased performance on the Wechsler Abbreviated Scales of Intelligence assessment (WASI) [30]. However, it is not clear whether the effect was due primarily to increased blood pressure versus an interaction between blood pressure and CKD.

The short-term effects on pediatric patients can manifest in various presentations—either subtle changes in symptoms or visible effects such as headache, seizures, or even change in mental status. When there have been chronic elevations in blood pressure, its effects on pediatric patients are ill defined, especially the cognitive implications. Lande et al. showed that patients with systolic blood pressures above the 95th percentile for age, height, and sex had significantly lower cognitive test scores for memory, attention, and concentration when compared to normotensive patients [3]. They went on to show that the use of antihypertensive medication could reverse cognitive dysfunction, which has also been demonstrated in the adult literature [20].

## 5. Discussion

Early diagnosis of HTN in pediatric patients may be important to minimize and/or correct cognitive dysfunction. With a multitude of effective treatments and good responses in children and adolescents with HTN, opportunities to recover or preserve cognitive functions are readily available. In adults, it has been shown that the use of antihypertensive medication can have a positive impact on cognitive function [20]. We hypothesize that antihypertensive treatment in pediatrics can have beneficial effects on cognitive function, but the guidelines for antihypertensive interventions and extent of cognitive benefits remain to be explored. Use of a standardized definition of hypertension such as that provided by the Fourth Report will better allow comparison between future studies in this area.

In the adult study by Elias et al., they showed that hypertensive adults performed poorer on a measure of speed, memory, spatial localization, simple motor skills, and learning [21]. Waldstein et al. also showed poorer scores for learning and memory in hypertensive adults [22]. Miller et al. demonstrated that the restorative effects of good blood pressure control may not be limited to just the cardiovascular system but have positive results in the brain [20]. They showed improvement of behavioral-cognitive scores with 15 months of antihypertensive treatment.

In pediatrics, the developing brain needs to be nurtured to ensure good development. From an early age, parents provide good nutrition, and stimuli and nurture their children to foster brain development. Efforts to minimize the harmful effects of HTN on the developing brain should be a priority in children and adolescents with HTN. Although children with CKD may have other contributory factors, it has been shown that children with hypertension have decreased cognitive performance [30, 31]. Studies have shown that hypertensive children and adolescents perform poorer on cognitive tests when compared to normotensive control subjects [3]. They also develop behavior and mood disturbances such as anxiety, depression, and problems with attention and have somatic complaints such as headaches and develop daytime fatigue along with difficulty with sleep initiation [5, 29]. Since an improvement in cognitive testing was seen in adults after antihypertensive treatment, it could be speculated that similar results would be seen in pediatric patients. If this is the case, then early and accurate diagnosis of pediatric HTN can provide opportunities to limit any possible cognitive dysfunction.

It has recently been shown that the combination of both lifestyle modification and medicines in children with HTN can result in improvement in cardiovascular target organ damage. Litwin et al. in 2010 showed reduction of left ventricular mass index and carotid intima-media thickness in 86 essential hypertensive children after 12 months of both nonpharmacological and pharmacological therapy [32]. Nonpharmacological therapies included increasing amount of physical activity and dietary changes. The benefit of such therapies on cognitive function and development in children is not yet clear but, given the profound impact of cognitive and cerebral function on adult outcomes and quality of life, these issues deserve further exploration in children.

## 6. Conclusion

Pediatric HTN and its effects on cognition and cognitive development are emerging as important concerns in children. There is an increasing body of literature that indicates that (1) HTN adversely affects cognitive function in children and adolescents and (2) effective treatment of HTN can reverse some of these cognitive impairments. There is evidence that deficits in cognition early in life adversely affect future learning and social interaction [7]. Development of an efficient and effective measurement tool for cognitive function will be instrumental to better detection of and intervention for cognitive impairment in pediatric hypertensive patients. More research is needed in this area because such findings may be significant and warrant further exploration into the consequences of HTN in pediatric patients. Future findings may have broad application to the areas of pediatric cognition, development, quality of life, and brain health.

## Figures and Tables

**Table 1:** Cognitive effects and conditions with increased incidence in pediatric hypertension.

Clinical symptoms
↓ Short-term memory
↑ Restlessness
↑ Temper tantrums
↑ Fatigue
↑ Sleep disturbances

Conditions

↑ ADHD
↑ ODD
↑ Depression
↑ Anxiety
